# The Ongoing Utility of lipoprotein lipase activity in diagnosing familial Chylomicronemia Syndrome

**DOI:** 10.1016/j.bbrep.2025.102245

**Published:** 2025-09-11

**Authors:** Gregorio Fariña, Magalí Barchuk, Amira Sleiman, Alejandro Castellanos Pinedo, Johnayro Gutierrez Restrepo, Valeria Zago, Juan Patricio Nogueira, Gabriela Berg

**Affiliations:** a Department of Clinical Biochemistry, Laboratory of Lipids and Atherosclerosis, Institute of Physiopathology and Clinical Biochemistry (INFIBIOC), Faculty of Pharmacy and Biochemistry, University of Buenos Aires, Buenos Aires, Argentina; b Faculty of Pharmacy and Biochemistry, University of Buenos Aires, National Scientific and Technical Research Council (CONICET), Buenos Aires, Argentina; c Santa Clara de Asís Hospital, Salta, Argentina; d San Jerónimo Hospital, Montería, Colombia; e University of Antioquia, Medellín, Colombia; f Center for Research in Endocrinology, Nutrition and Metabolism, Faculty of Health Sciences, National University of Formosa, Formosa, Argentina

**Keywords:** Lipoprotein lipase activity, Familial chylomicronemia syndrome, Diagnosis, Multifactorial chylomicronemia syndrome, Severe hypertriglyceridemia

## Abstract

**Objective:**

Given that lipoprotein lipase (LPL) activity assays are not standardized for clinical use, we aimed to define reference values applicable to our clinical setting and identify a cut-off point to help distinguish Familial Chylomicronemia Syndrome from Multifactorial Chylomicronemia Syndrome, particularly in patients with inconclusive genetic findings.

**Methods:**

We evaluated 28 patients with a history of TG levels above 880 mg/dL (10 mmol/L), and assessed their likelihood of FCS using the Moulin score. LPL activity was measured in post-heparin plasma using a radiometric assay. Thirty normotriglyceridemic controls were used to define reference values. Genetic testing for FCS canonical genes and lipid profile was performed in all sHTG patients.

**Results:**

The reference value for LPL activity was 33.3 (18.7–70.3) mIU, with a cut-off of 8.42 mIU (25 % of the median of NTG) to distinguish FCS from MCS. Eighteen patients without genetic variants in canonical genes, a Moulin score <9 and LPL activity >25 % of NTG, were classified as MCS. Five genetic diagnosed FCS patients, with a Moulin score>10 presented LPL activity <25 % of NTG. Four patients with inconclusive genetic results and a Moulin score>10 were classified as FCS according to LPL activity.

**Conclusion:**

LPL activity in patients with sHTG could be useful for differentiating FCS and MCS, particularly in patients with ambiguous or negative genetic findings, highlighting the need for specialized laboratory support in diagnostics.

## Highlights

• LPL activity helps distinguish FCS from multifactorial causes of sHTG.
• LPL cut-off value of 25 % of the median of NTG identifies FCS patients with high accuracy.
• LPL activity aids diagnosis when genetic results are inconclusive.
• Specialized lab support is crucial for accurate FCS diagnosis.

## 1 Introduction

Severe hypertriglyceridemia (sHTG), defined as a triglyceride (TG) concentration greater than 880 mg/dL (10 mmol/L), is a rare condition, that includes familial chylomicronemia syndrome (FCS) in some cases, and more frequently oligogenic multifactorial chylomicronemia syndrome (MCS) [1].

FCS is an autosomal recessive disorder (1–3 patients in 1,000,000 inhabitants), characterized by sHTG, resulting from a deficit in the catabolism of chylomicrons (CM) and their subsequent accumulation in plasma [1]. The main responsible for the catabolism of CM is lipoprotein lipase (LPL), an enzyme that is anchored to the capillary endothelium of the tissue that synthesizes it, through a bond that can be released by heparin, and hydrolyses TG contained in this lipoprotein, generating free fatty acids (FFA). Although LPL also hydrolyses TG from endogenous synthesis that are transported by very low-density lipoprotein (VLDL), the lack of LPL activity primarily affects the catabolism of CM and secondarily VLDL [2]. The accumulation of these large and loaded TG particles in the circulation causes milky serum in patients with this disease, along with characteristic clinical manifestations such as eruptive xanthomas on the trunk and extremities, lipemia retinalis, recurrent abdominal pain, hepatosplenomegaly and pancreatitis (acute or recurrent), the latter being the main cause of morbidity and mortality in these patients [3].

The activity of LPL depends not only on the correct expression of the encoding gene but also on various activators and inhibitors. Among the factors that positively regulate LPL gene expression are insulin and peroxisomal proliferator-activated receptors (PPAR), especially PPAR-γ. However, post-translational regulation, which is mediated by different activators and inhibitors, is highly significant. To the date, four main activators are recognized: apoprotein (apo) AV, apoCII, glycosylphosphatidylinositol-anchored high-density lipoprotein-binding protein 1 (GPIHBP1), and lipase maturation factor 1 (LMF1) [4]. Among the inhibitors, apoCIII and angiopoietin-like proteins type 3 and 4 (ANGPTL 3 and 4) are the most significant [4,5]. Given this important post-translational regulation, the measurement of LPL concentrations does not completely reflect its final activity [2,6,7].

The most frequent genetic alterations in FCS are loss-of-function (LOF) mutations in the *LPL* (60–80 %), *APOC2* (5 %), *APOA5* (10 %), *LMF1* (1 %) and *GPIHBP1* (5 %) genes [8]. However, these data come from studies carried out in European populations, with few evaluations of the prevalence of FCS or the most frequent mutations in Latin America [9,10].

Importantly, even after performing a directed genetic study, in approximately 30 % of patients, the causal variant is not found, which makes it difficult to diagnose FCS and differentiate it from the other primary cause of sHTG, the MCS [11]. This latter syndrome includes patients with polymorphisms that affect genes involved in the synthesis or catabolism of TG-rich lipoproteins (TRL), or patients who present heterozygosity for any of the five canonical genes of FCS [3].

Given the difficulty of diagnosis this pathology, different scoring systems have been proposed that attempt to identify probable, improbable or very improbable cases, among which the Moulin [12], Brisson [13], and North American FCS (NAFCS) [14] scores stand out. Although these scores are indicative, they are not conclusive and, in some cases, present contradictory results with genetics. In this context, the measurement of LPL activity is a complementary diagnostic tool to a comprehensive clinical-biochemical-genetic approach. Furthermore, the assessment of LPL activity to confirm the pathogenicity of new variants or genes not yet described is crucial.

In vitro assays for assessing LPL activity in post-heparin plasma (PHP) were developed many years ago. These are complex manual procedures, which employ different substrates and detection methods, inhibitors of other lipases and are dependent on the diagnostic laboratory experience [15]. Despite these limitations, these methods are still considered the gold standard for the evaluation of enzymatic activity [16]. Hence, as reference values depend on the complexity of the method and the diversity of conditions used, each laboratory should establish its own, taking into account that the normal LPL activity intervals may not be interchangeable. In this regard, it is important to highlight that, if LPL activity is used for FCS diagnosis, it is necessary to standardize the methodologies and cut-off values [17,18].

The objective of this study was, first, to determine LPL activity from normotriglyceridemic (NTG) patients, in order to establish our laboratory's own reference values, and a cut-off point to differentiate between FCS and MCS patients. Second, we aimed to study PHP-LPL activity, as a complementary diagnostic tool, for FCS in our population, mainly in patients with inconclusive genetic results.

## 2 Materials and methods

1. **Patients and clinical data:** twenty-eight subjects with sHTG and suspected FCS were evaluated, after verifying the absence of secondary causes of sHTG (e.g. diabetes mellitus prior to HTG, alcoholism, chronic kidney disease, lipodystrophy, hypertriglyceridemic medication, etc.), who had been referred to our laboratory by medical professionals from different regions of Argentina and Colombia. All patients underwent genetic testing for mutations in five canonical genes (*LPL, APOC2, APOA5, GPIHBP1,* and *LMF1*). The Moulin score was calculated, and patients were initially classified as very likely FCS (Moulin score ≥10), unlikely FCS (Moulin score ≤9), and very unlikely FCS (Moulin score ≤8) [12]. Written consent was obtained from all participants included in the study, which was approved by the Ethics Committees of the Faculty of Pharmacy and Biochemistry of the University of Buenos Aires (RESCD-2021-842-E-UBA-DCT-FFYB).

*Population for LPL reference values*: A group of 30 healthy, NTG adult subjects (33 ± 5 years, 68 % male) was recruited as a control group, to determine the normal range of PHP-LPL activity. All participants attended the Lipids and Atherosclerosis Laboratory of the José de San Martín Clinical Hospital, between December 2021 and December 2022, and personal and family risk factors were assessed via self-report questionnaires. The following exclusion criteria were applied: alcohol consumption >20 g/day; personal or family history of diabetes, overweight or obesity, cardiovascular disease (CVD), hypothyroidism; recent history of acute illness, renal disorders; seropositive hepatitis B or C; or other endocrine disorders such as polycystic ovaries. None of the control subjects received corticosteroids, hormonal therapies, immunosuppressive agents, or drugs that influence lipid metabolism, such as statins or fibrates.

Additionally, a receiver operating characteristic (ROC) curve was constructed for 23 patients with confirmed FCS and 33 patients with confirmed MCS by genetic testing to establish sensitivity, specificity, and cut-off points for identifying FCS patients.

In all groups, the weight and height of each participant were measured, and body mass index (BMI) was calculated to evaluate the degree of obesity.

- Samples: After 12-h fast, blood samples were drawn prior to heparin injection. Serum total cholesterol (TC), TG, HDL-cholesterol (HDL-C), and ApoB were assessed.

To measure LPL activity, heparin (60 IU/kg of body weight) was administered intravenously. After 10 min, blood was collected from the contralateral arm in tubes on ice. PHP was obtained by centrifugation at 1500*g* at 4 °C for 10 min and kept at −70 °C until LPL activity analysis.

2. **Biochemical determinations**: TC, HDL-C and TG were measured via commercial enzymatic kits (Mindray, China) on a Mindray BS-360 E autoanalyzer; the intra-assay coefficient of variation (CV) were 0.9 and 0.8 % respectively, and the inter-assay CV were 1.4 and 1.6 %, respectively. Serum ApoB was determined by immunoturbidimetry (Mindray, China); with an intra-assay CV of 1.5 % and an inter-assay CV of 2.4 %.

## 3 Lipoprotein lipase activity

LPL activity was determined in PHP by measuring the oleic acid produced by the enzyme-catalyzed hydrolysis of a radiolabelled substrate, according to a modified Nilsson-Ehle method [19].

### 3.1 Substrate preparation

The substrate for determining PHP-LPL activity was prepared as an emulsion containing labelled [9,10-3H(N)]-Triolein (ARC ART-199, 1 mCi/ml) and unlabelled triolein (Sigma T-7140) with a final concentration of 1.3 mmol/ml glycerol trioleate, specific activity 10 × 10^6^ cpm/mmol, mixed with 0.11 mmol/ml of l-lysophosphatidylcholine (Sigma L-4129) used as emulsifier and 4 % bovine serum albumin (Sigma A- 6003), the latter added in order to capture FFA released. This mixture was sonicated for 6 cycles of 30 s each to form micelles resembling lipoproteins, in a Vibra-cell sonicator in 0.2 M Tris-HCl buffer pH 8.0 with 0.3 M NaCl.

### 3.2 Substrate preparation for a functional assay in patients with non-LPL mutations

Two substrates for determining PHP-LPL activity were prepared in parallel, one with saline solution and another with the addition of 10 % v/v normolipemic human serum, inactivated at 56 °C for 30 min, as an exogenous source of enzymatic cofactors such as apoCII and apoAV. The latter constitutes an in vitro functional assay to rule out the absence of the mentioned cofactors [16].

### 3.3 Sample incubation and released FA extraction

Substrates were incubated in parallel with PHP 1:10 in saline solution to determine total lipolytic activity (TLA), and simultaneously with PHP 1:5 in saline solution and NaCl 1 M, which acts as an LPL inhibitor, to assess the remaining lipolytic activity (RLA) during 45 min at 37 °C. After incubation, the reaction was stopped on ice and the released FA were isolated via extraction with a methanol/chloroform/heptane mixture (1.45:1.25:1 by volume) and carbonate-borate buffer, pH 10.5. The released ^3^H-FA were quantified in a liquid scintillation analyzer (HIDEX 300 SL).

### 3.4 LPL activity calculation

The LPL activity of each individual was calculated as the difference between TLA and RLA. The enzymatic activity was expressed in milli (m) international units (mIU, as 1 IU = 1 μmol of fatty acid released per minute) per ml of PHP.

### 3.5 Analytical performance

To evaluate the analytical performance of LPL activity measurement, first we assessed the optimal substrate concentration. Besides, the limit of detection (LOD), limit of quantification (LOQ), intra-assay and inter-assay coefficients of variation (CVs) were calculated. The LOD was determined by analyzing the lowest concentration of LPL activity that could be reliably distinguished from the background noise, while the LOQ was determined as the lowest concentration at which LPL activity could be quantified with acceptable precision and accuracy based on a signal-to-noise ratio of 10:1. Intra-assay CV was determined by performing multiple replicates of the same sample within a single run, and inter-assay CV was calculated from 20 successive assays of the same sample.

4. **Genotyping:** In patients with suspected FCS, a saliva sample was obtained and submitted for exome sequencing and subsequent analysis of prioritized variants in the gene panel (*LPL, APOC2, APOA5, GPIHBP1, LMF1, APOE*) by paired-end synthesis using the NovaSeq Sequencing System (Illumina) platform, suitable for diseases with a Mendelian inheritance pattern (monogenic), with a high genotype-phenotype association. If no variants that met these conditions were found, the report was considered negative. The analysis of the variants was generated on the basis of the information available in biological databases and scientific publications.
5. **Statistical analysis**: Data are presented as mean ± standard deviation (SD) or median (range) according to a normal or skewed distribution, respectively. The data distribution was tested by the Kolmogorov-Smirnov test. Differences between groups were tested using χ^2^, Mann-Whitney test, based on the data distribution, controlling for necessary confounders. For the comparison between paired samples, a Wilcoxon signed-rank test was performed. Spearman analysis, for nonparametric variables, was used to determine correlations between parameters. A receiver operating characteristic (ROC) curve for LPL activity was constructed to diagnose FCS, using genetic data as the gold standard, and a cut-off point was established according to the Youden index. The SPSS 19.0 (Chicago, USA) and GraphPad Prism 5.01 (La Jolla, USA) software packages were used for statistical analysis. A two-tailed p < 0.05 was considered significant.

## 4 Results

- Reference Values for LPL Activity

The reference values for LPL activity were obtained from the NTG group in our laboratory. It primarily consisted of men, aged 21–43 years. Although there was a difference in the sex distribution of the population, the pattern of biochemical parameters, as well as LPL activity, were similar across groups, so it was unified for further analysis. Regarding lipid and lipoprotein profiles, no alterations were verified, thereby validating this population as normolipemic. Moreover, no personal or familial risk factors were identified in NTG participants (Additional file 1).

The reference value for LPL activity obtained from this group was 33.3 (18.7–70.3) mIU (median (range)), with a confidence interval (CI95) of 28.5–41.3. The intra-assay CV was 5.1 %, whereas the inter-assay CV, was 13.2 %. Because of the complexity of this assay, the CVs are considered acceptable. The calculated LOD was 0.144 mIU, and the limit of LOQ was determined to be 1.83 mIU. The evaluation of optimal substrate concentration can be found in Additional file 2.

**Fig. 1:**
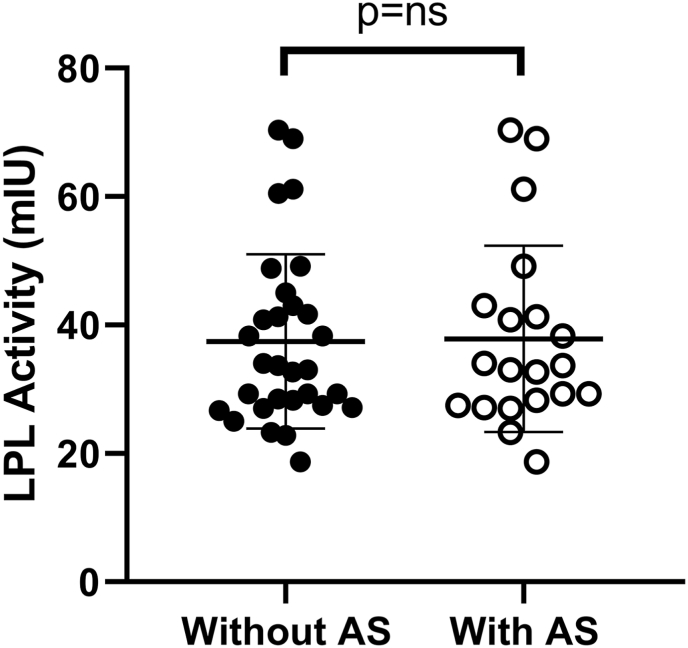
LPL activity of reference group with and without the addition of cofactors. LPL: lipoprotein lipase; AS: activating serum. P = 0.544, U Mann Whitney test.

For further evaluation of ApoAV or ApoCII deficiency, we performed a functional assay in the NTG population to verify that the enzyme activity remained unchanged with the addition of normal human serum, so a single reference value was established regardless of the addition of cofactors (Fig. 1).

- ROC curve and cut-off point

**Fig. 2:**
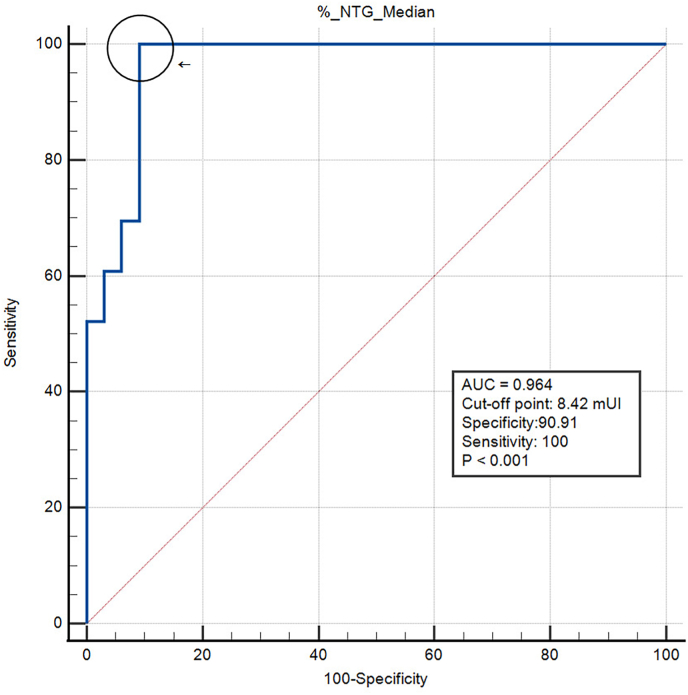
ROC curve for the Lipoprotein lipase activity.

The ROC curve for the LPL activity determined by the method described in this work is shown in Fig. 2. The area under the curve (AUC) was 0.964 (CI95 = 0.877–0.996), and the best cut-off points for distinguishing MCS from FCS were 6.26 mIU and 8.42 mIU, corresponding to 20 % and 25 % of the median activity of NTG respectively, with a specificity of 91 % in both cases, and a sensitivity of 70 % and 100 %, respectively, according to the maximum Youden index. The positive predictive value for 6.26 mIU was 0.79, and for 8.42 mIU, it was 0.8. The negative predictive values for 6.26 mIU was 0.88 and for 8.42 mIU was 1. The construction of the ROC curve is shown in Additional file 3.

- LPL activity, molecular diagnosis and clinical features of sHTG patients

**Fig. 3:**
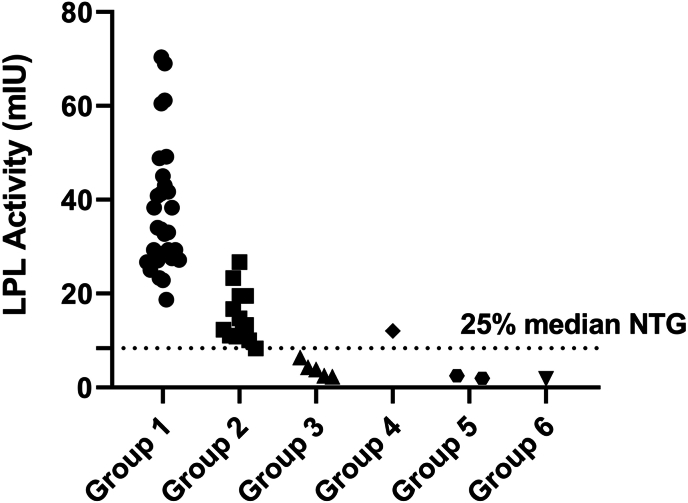
Lipoprotein lipase activity for assessed patients. LPL: lipoprotein lipase, NTG: normotriglyceridemic. Group 1: NTG Controls; Group 2: Multifactorial Chylomicronemia Syndrome (MCS); Group 3: Familial Chylomicronemia Syndrome (FCS) (Monogenic confirmed pathogenic variants); Group 4: MCS (LPL heterozygous variant); Group 5: FCS (Heterozygous Cofactor Variants); Group 6: FCS (Compound Heterozygous).

Table 1 describes the clinical, biochemical and genetic characteristics of the studied patients, as well as LPL activity. Additionally, Fig. 3 shows the LPL activity in all groups.

**Table 1:** Clinical, biochemical, and genetic profile of the assessed patients.

Group		Age (median, range)	BMI (median, range)	Moulin Score (median, range)	Pancreatitis (n/N)	Diabetes (n/N)	Lipid lower therapy (n/N)	TG (mg/dl)	ApoB (mg/dl)	TG/ApoB	TG/TC	LPL (mIU)	% NTG median	Variant
1	Control (n = 30)	33 (21–43)	23.6 (21.2–25.3)	–	0/30	0/30	0/30	85 (39–142)	84 (53–143)	1.0 (0.7–1.1))	0.5 (0.3–0.6)	33.3 (18.7–70.3)	–	–
2	MCS (n = 18)	38 (21–50)	28.8 (24.0-30-8)	8 (7–9)	4/18	10/18	5/10	803 (374–1873)	120 (82–152)	4.6 (3.2–8.7)	2.5 (1.9–3.8)	14.31 (12.55–18.31)	42.9 (37.6–54.9)	*-No variants identified in the molecular study (n* = *16)*
														*-APOE: c.526C* > *T (p.*Arg*1*76Cys*) (n* = *1)*
														*-APOE: c.388T* > *C (p.Cys130Arg) (n* = *2)*
3	HTG with homozygous in LPL or APOA5 (n = 5) FSC	49 (33–57)	26.4 (23.9–28.1)	12 (11–13)	5/5	2/5	0/5	2230 (967–2925)	79 (52–82)	27.2 (11.9–55.2)	10.0 (4.6–12.7)	3.65 (3.19–5.24)	10.9 (9.6–15.7)	*APOA5*: c.694 T > C; p.Ser232Pro (P) (n = 1)
														*LPL* c.644G > A; p.Gly215Glu, homozygous, (P) (n = 3)
														*LPL*: c.245G > A; p.Trip82Ter homozygous. (PP) (n = 1)
4	HTG with heterozygous in *LPL* (n = 1) MCS	54	24.5	9	1/1	0/1	0/1	2169	213	10.2	3.3	12.00	36.0	*LPL* c.106G > A, p.Asp36Asn, heterozygous (PB)
5	HTG with heterozygous in APOC2 (n = 1) or *APOA5* (n = 1)	59	25.8	12	1/1	1/1	1/1	753	98	7.7	4.6	2.50	7.5	*APOC2* c.162C > T p.Ala54Ala heterozygous (PB)
														*ApoE* p.Cys130Arg, heterozygous (VUS)
		58	26.2	11	0/1	0/1	0/1	820	110	7.5	3.5	1.94	5.7	*APOA5* c.56C > G, p.Ser19Trp heterozygous (VUS)
6	HTG with heterozygous in LPL and APOA5 (n = 2)	47	36.0	12	1/1	1/1∗	1/1	954	105	9.1	5.6	<1.83	<5.49	*LPL* c.953 A > G, p.Asn318Ser (VUS)
		48	37.0	10	1/1	1/1∗	0/1	879	90	9.8	5.2			*APOA5* c.56C > G p.Ser19Trp (VUS) compound heterozygotes

BMI: Body mass index; n/N: absolute frequency; TG: triglycerides; ApoB: apolipoprotein B; TC: total cholesterol; LPL: lipoprotein lipase. PB: probable benign, VUS: Variant of uncertain significance, PP: probable pathogenic, P: pathogenic. ∗Post hypertriglyceridemia.

Group 1 shows the NTG subjects and Group 2 includes MCS patients. The latter consists of 18 patients without genetic variants in canonical genes, some of whom have mutations in *APOE.* In all these patients LPL activity was higher than 25 % of that reported for NTG, and Moulin score was less than 9.

Group 3 includes 5 genetically diagnosed FCS patients, three of them with previously described pathogenic *LPL* variants (p.Gly215Glu) and one of them in *APOA5* (p.Ser232Pro); the last patient presented a non-previously reported variant in *LPL* (p.Trp82Ter) which, as a consequence of the LPL activity, was considered as probable pathogenic. In all these cases, Moulin score was higher than 11 and LPL activity was below the cut-off point of 8.42 mIU, with an activity percentage below 10.9 (9.6–15.7).

Group 4 depicts a patient with heterozygosity in *LPL* (p.Asp36Asn), a Moulin score of 8 and LPL activity over 25 % of NTG, on the basis of these findings, the patient was classified as MCS. Group 5 includes two heterozygous patients, one in *APOC2* (p.Ala54Ala) and the other in *APOA5* (p.Ser19Trp), with Moulin scores of 12 and 11 and LPL activities of 2.5 and 1.8 mIU (7.5 and 5.5 % of NTG), respectively. Even though the genetic results were inconclusive, according to the Moulin score and LPL activity, both patients were classified as FCS.

Group 6 shows two compound heterozygous sibling patients, with *LPL* (p.Asn318Ser) and *APOA5* (p.Ser19Trp) mutations. Moulin score was greater than 10 and LPL activity was lower than 1.83 mIU, consequently these strains were classified as FCS.

**Fig. 4:**
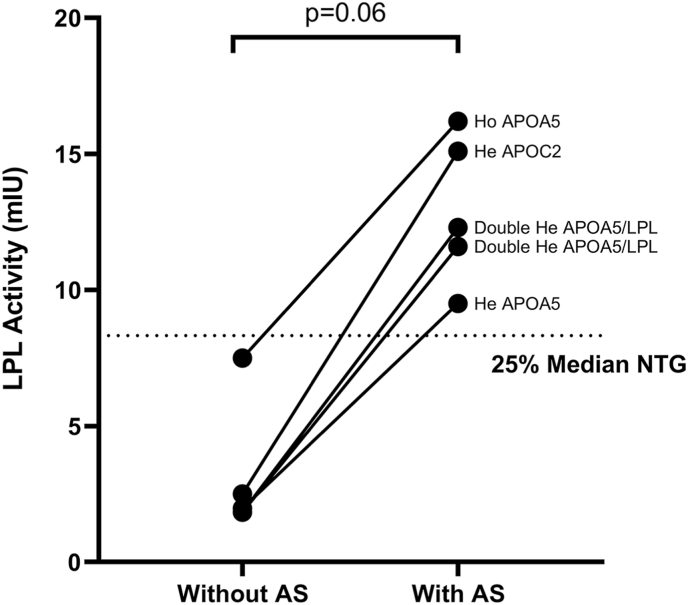
In vitro functional assay. Addition of AS increases LPL activity in cases of cofactors deficiency. LPL: lipoprotein lipase; AS: activating serum; Ho: homozygous; He: heterozygous. Wilcoxon rank-test.

In patients with variants in *APOA5* and *APOC2*, a functional assay was performed. Although only a statistical trend was noted, the addition of activating serum increased the in vitro activity of LPL displaying normal values and confirming the normal LPL function in the presence of cofactors (Fig. 4).

**Fig. 5:**
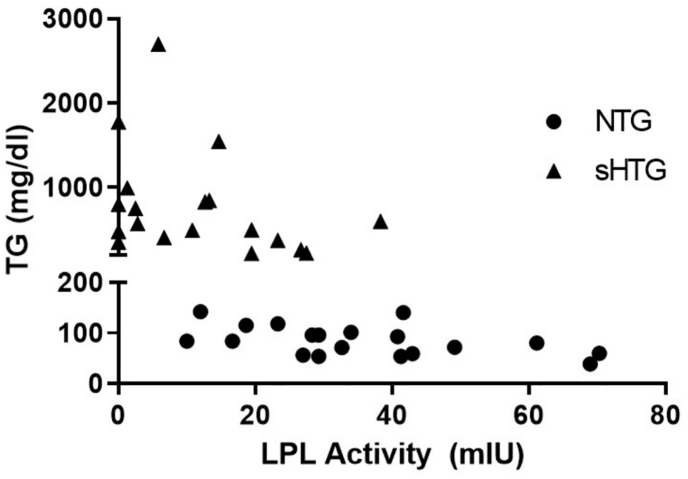
Correlation between TG and LPL activity. TG: triglycerides; LPL: Lipoprotein lipase; NTG: normotriglyceridemic; sHTG: severe hypertriglyceridemic.

With respect to the correlation between LPL activity and TG levels, among NTG controls, a trend was observed (r = −0.4260, p = 0.07), which was significant when MCS and FCS patients were included in the statistical analysis (r = −0.5831, p < 0.01) (Fig. 5).

## 5 Discussion

In this study, we report a reference value for LPL activity and establish a diagnostic cut-off point to facilitate the identification of FCS patients in our region. Additionally, we present cases of patients with sHTG, demonstrating how LPL activity measurements can enhance diagnostic accuracy. Diagnosing FCS remains challenging, and delays in distinguishing it from MCS significantly affect the quality of life of patients. This delay increases the risk of recurrent pancreatitis and often leads to prolonged, fragmented care across multiple healthcare providers [20].

On the basis of clinical suspicion, the definitive diagnosis of FCS is provided by a confirmatory genetic analysis of homozygous or double heterozygous mutations for the five canonical genes. Although it is the gold standard, the use of genetics as the only diagnostic tool offers a limited perspective on FCS [11]. In this regard, in many patients, genetic testing is not conclusive even with a Moulin score that indicates very probable FCS. Moreover, in the APPROACH study, 21 % of the patients did not have any defined genetic variants but were recruited because of their clinical phenotype and LPL activity below 20 % of their normal population [21].

Therefore, in these cases, the measurement of LPL activity has emerged as an accurate diagnostic tool [18,21], although the methods for its measurement are often difficult to standardize, given the high heterogeneity that exists between techniques and laboratories [[14], [15], [16]]. This highlights the need to validate the assay and establish reference values for the NTG population, to propose an activity percentage to aid in completing the diagnosis of FCS. In our study, the cut-off value with higher sensitivity and specificity corresponded to 25 % of the median LPL activity in NTG patients (8.42 mIU). The previously suggested cut-off value of 20 % of the median NTG LPL activity [21], in our case (6.26 mIU), presented the same specificity but lower sensitivity, and thus lower negative predictive value, thus, its use could result in some patients being undiagnosed in our population. In addition, our study revealed lower LPL activity in FCS patients than in MCS and NTG patients. This is an expected result, which validates our assay, in contrast to previous studies in which contradictory findings revealed higher LPL activity in the MCS group than in the NTG group [17]. When comparing the different methods, it is important to highlight that all of them are in-house assays. Some use human VLDL as a substrate, which could, if not well assessed, introduce variability [16]; while others define their cut-off values based on a population with MCS [17]. These differences clearly indicate that the methods are not interchangeable. However, each laboratory can choose the approach that best suits its resources and expertise, as long as the method is properly validated and that appropriate cut-off values are established.

The addition of normal human serum as a source of cofactors (apoCII and apoAV) in the in vitro functional assay helps to reveal the absence of these proteins [16]. In our study, in the NTG controls, there were no differences in the enzymatic activity with or without the addition of cofactors, which allowed the establishment of a single reference value for LPL activity. However, in patients with mutations in *APOC2* and/or *APOA5*, an increase in LPL activity was observed with the addition of normal human serum, which is consistent with the genetic results, and represents an in vitro validation of the mutations.

In those patients with homozygous or compound heterozygous pathogenic and probably pathogenic mutations in *LPL* and *APOA5*, LPL activity was below the cut-off point of 8.6mIU, in accordance with previous reports [18,21]. In addition, as formerly stated [18], LPL activity has been poorly described in single heterozygous *LPL* as well as *APOA5* mutation carriers, and most of these patients have normal or mildly increased TG levels under metabolic control, probably due to residual LPL activity. Nevertheless, in our study, in one patient with a heterozygous apparently benign mutation in *APOC2*, one patient with a heterozygous variant of unknown significance (VUS) in *APOA5*, and two patients with double heterozygous mutations of unknown significance in *LPL* and *APOA5*, LPL activity was quite below the cut-off point of 8.6 mIU. Besides, these last two patients were siblings, and presented the same variants in *LPL* and *APOA5*.

The variant in *APOA5* 56C > G (p.Ser19Trp), although considered a VUS, is a well-known polymorphism in most populations, with minor allele frequencies ranging from 5 % to 15 % [22]. Even though some authors [23] consider that this is not associated with FCS, there is limited evidence supporting the cosegregation of *APOA5* variants with hypertriglyceridemia in multigenerational families. In this context, our results strengthen the evidence for the pathogenicity of this *APOA5* variant, in line with recent publications [24], and underscore the need for further investigation into these findings. We also suggest that screening relatives, when clinically appropriate, is essential. The variation in LPL activity among *APOA5*-deficient patients highlights the complexity of the phenotype, emphasizing the importance of assessing LPL activity to better understand the significant phenotypic variability observed in these individuals.

As we previously reported [25,26], as well as others [24], the presence of a Moulin Score above 10 and LPL activity below a validated cut-off point, in the presence of a novel variant or a VUS, provides strong evidence supporting the pathogenicity of a variant.

The c.953 A > G (p.Asn318Ser) mutation in *LPL* has been described and is associated with a lack of LPL activity [27]. Moreover, the four patients presented a Moulin score compatible with FCS, and in these cases LPL activity completed the diagnosis and was in accordance with the clinical findings. Overall, these findings provide a clear explanation for the severe phenotype in compound heterozygous patients, attributed to the additive effects of the mutations.

Regarding the clinical characteristics of FCS, BMI has consistently been reported to be lower than that of MCS. In data from the APPROACH and COMPASS trials, BMI <26.1 kg/m^2^ was used to differentiate between FCS and MCS [14]. A recent multicenter study involving 151 patients (75 FCS and 76 MCS) revealed that a BMI was a positive predictor of FCS [8]. However, in our study, we found that 2 probable FCS patients presented a BMI higher than 30 kg/m^2^, and some of the patients presented with diabetes mellitus, as reported in previous studies [28]. The increased prevalence of diabetes resulting from recurrent acute pancreatitis has been described [29]; a recent study revealed a high 10-year incidence of diabetes in young adults with MCS and FCS compared with the general population [30]. Therefore, BMI should be carefully considered in the evaluation of FCS, especially in cases of late diagnosis and after recurrent acute pancreatitis episodes that could have triggered the development of diabetes [30].

One limitation of our study is the small number of patients. However, the low prevalence of this condition should be considered. Additionally, genetic testing focused only on the five canonical genes, and some patients may carry other mutations that were not evaluated. In addition, neither autoantibodies nor polygenic scores for TG levels were evaluated in any patient within this cohort, preventing us from addressing these potential forms of autoimmune or genetic susceptibility. Although other cohorts reported that FCS patients have a younger age at diagnosis, lower BMI and a higher prevalence of pancreatitis [31], it should be considered that all of our patients were diagnosed during adulthood. This delay was partly due to the low frequency of the disease, disparities in healthcare access and lack of awareness among physicians.

Another limitation of this study is the limited number of patients with a definitive diagnosis of FCS based on genetic criteria. As more patients with confirmed diagnoses are identified, it will be necessary to reassess the proposed LPL activity cut-off value to ensure its robustness. Until then, the current results should be interpreted with caution and regarded as exploratory, warranting further validation in larger, independent cohorts.

Overall, the available data and our experience demonstrate that measuring plasma LPL activity in patients with sHTG could serve as an additional tool to differentiate FCS from MCS, particularly in cases where mutations in the target genes are not detected or where the results are inconclusive. In this study, we show that a cut-off value of 25 % for LPL activity serves as an effective complementary diagnostic tool for FCS, providing excellent sensitivity and specificity. This offers a valuable diagnostic option in situations where genetic testing yields inconclusive results. Further meta-analysis and multicenter studies will allow to compare results among different populations.

## CRediT authorship contribution statement

**Gregorio Fariña:** Writing – original draft, Methodology, Investigation, Formal analysis, Data curation, Conceptualization. **Magalí Barchuk:** Writing – review & editing, Writing – original draft, Methodology, Investigation, Formal analysis, Data curation. **Amira Sleiman:** Writing – original draft, Investigation. **Alejandro Castellanos Pinedo:** Writing – original draft, Investigation. **Johnayro Gutierrez Restrepo:** Writing – original draft, Investigation. **Valeria Zago:** Writing – review & editing, Methodology, Investigation. **Juan Patricio Nogueira:** Writing – review & editing, Writing – original draft, Investigation. **Gabriela Berg:** Writing – review & editing, Writing – original draft, Validation, Supervision, Project administration, Methodology, Investigation, Formal analysis.

## Declaration of competing interest

The authors declare that they have no known competing financial interests or personal relationships that could have appeared to influence the work reported in this paper.

## Data Availability

Data will be made available on request.
