# Supplementary material for: The Ongoing Utility of lipoprotein lipase activity in diagnosing familial Chylomicronemia Syndrome
Source: Biochem Biophys Rep. 2025 Sep 11;44:102245. doi: 10.1016/j.bbrep.2025.102245 (PMC12803793; doi:10.1016/j.bbrep.2025.102245)
Supplement: Multimedia component 1 [file mmc1.docx]

**Additional File 1. Clinical and biochemical characteristics of the NTG population.**

|  | **Mean** | **SD** | **Median** | **Minimum** | **Maximum** |
| --- | --- | --- | --- | --- | --- |
| **Age (years)** | 33 | 5 | 33 | 21 | 43 |
| **BMI (Kg/m^2^)** | 23.3 | 1.4 | 23.6 | 21.2 | 25.3 |
| **Total cholesterol (C) (mg/dl)** | 180 | 28 | 179 | 129 | 230 |
| **Triglycerides (mg/dl)** | 85 | 31 | 80 | 39 | 142 |
| **HDL-C (M) (mg/dl)** | 52 | 10 | 51 | 40 | 77 |
| **HDL-C (W) (mg/dl)** | 80 | 15 | 81 | 62 | 97 |
| **LDL-C (mg/dl)** | 99 | 26 | 99 | 64 | 156 |
| **Non HDL-C (mg/dl)** | 106 | 32 | 113 | 14 | 159 |
| **RLP-C (mg/dl)** | 13 | 8 | 14 | 2 | 29 |
| **ApoB (mg/dl)** | 84 | 23 | 80 | 53 | 143 |

NTG: normotriglyceridemic; BMI: Body mass index; C: Cholesterol; M: Male; RLP: Remnant lipoproteins; W: Women; WC: Waist circumference.
