# Supplementary figures and images for: The Ongoing Utility of lipoprotein lipase activity in diagnosing familial Chylomicronemia Syndrome
Source: Biochem Biophys Rep. 2025 Sep 11;44:102245. doi: 10.1016/j.bbrep.2025.102245 (PMC12803793; doi:10.1016/j.bbrep.2025.102245)

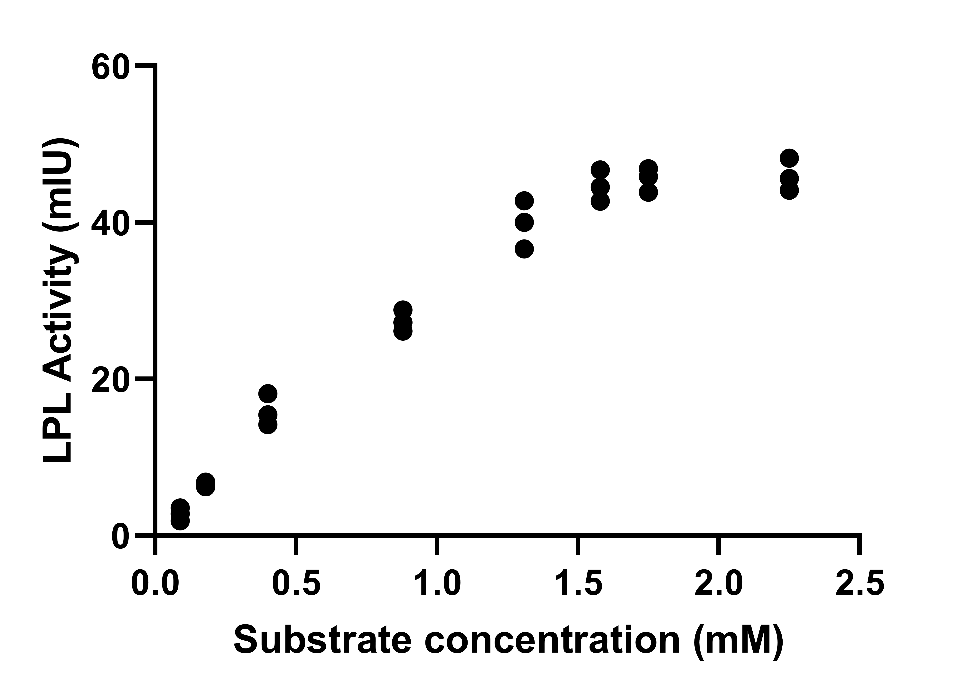
**Additional File 2. Optimal substrate concentration.**

LPL: Lipoprotein lipase

Supplement: Multimedia component 2 [file mmc2.docx]
