# Supplementary material for: The Ongoing Utility of lipoprotein lipase activity in diagnosing familial Chylomicronemia Syndrome
Source: Biochem Biophys Rep. 2025 Sep 11;44:102245. doi: 10.1016/j.bbrep.2025.102245 (PMC12803793; doi:10.1016/j.bbrep.2025.102245)
Supplement: Multimedia component 3 [file mmc3.docx]

**Additional file 3. ROC curve construction.**

| Criterion | Sensitivity | 95% CI | Specificity | 95% CI | Youden Index |
| --- | --- | --- | --- | --- | --- |
| <4 | 0 | 0,0 - 14,8 | 100 | 89,4 - 100,0 | 0 |
| ≤4 | 4,35 | 0,1 - 21,9 | 100 | 89,4 - 100,0 | 0,0435 |
| ≤6,5 | 8,7 | 1,1 - 28,0 | 100 | 89,4 - 100,0 | 0,087 |
| ≤7,11 | 13,04 | 2,8 - 33,6 | 100 | 89,4 - 100,0 | 0,1304 |
| ≤7,5 | 17,39 | 5,0 - 38,8 | 100 | 89,4 - 100,0 | 0,1739 |
| ≤8,43 | 21,74 | 7,5 - 43,7 | 100 | 89,4 - 100,0 | 0,2174 |
| ≤9 | 26,09 | 10,2 - 48,4 | 100 | 89,4 - 100,0 | 0,2609 |
| ≤9,05 | 30,43 | 13,2 - 52,9 | 100 | 89,4 - 100,0 | 0,3043 |
| ≤9,57 | 39,13 | 19,7 - 61,5 | 100 | 89,4 - 100,0 | 0,3913 |
| ≤9,7 | 43,48 | 23,2 - 65,5 | 100 | 89,4 - 100,0 | 0,4348 |
| ≤10,58 | 47,83 | 26,8 - 69,4 | 100 | 89,4 - 100,0 | 0,4783 |
| ≤10,96 | 52,17 | 30,6 - 73,2 | 100 | 89,4 - 100,0 | 0,5217 |
| ≤11,5 | 52,17 | 30,6 - 73,2 | 96,97 | 84,2 - 99,9 | 0,4914 |
| ≤11,54 | 56,52 | 34,5 - 76,8 | 96,97 | 84,2 - 99,9 | 0,5349 |
| ≤12,26 | 60,87 | 38,5 - 80,3 | 96,97 | 84,2 - 99,9 | 0,5784 |
| ≤15,56 | 60,87 | 38,5 - 80,3 | 93,94 | 79,8 - 99,3 | 0,5481 |
| ≤15,71 | 65,22 | 42,7 - 83,6 | 93,94 | 79,8 - 99,3 | 0,5916 |
| ≤17,5 | 69,57 | 47,1 - 86,8 | 93,94 | 79,8 - 99,3 | 0,6351 |
| ≤20 | 69,57 | 47,1 - 86,8 | 90,91 | 75,7 - 98,1 | 0,6048 |
| ≤20,08 | 73,91 | 51,6 - 89,8 | 90,91 | 75,7 - 98,1 | 0,6482 |
| ≤20,92 | 78,26 | 56,3 - 92,5 | 90,91 | 75,7 - 98,1 | 0,6917 |
| ≤22 | 82,61 | 61,2 - 95,0 | 90,91 | 75,7 - 98,1 | 0,7352 |
| ≤22,08 | 86,96 | 66,4 - 97,2 | 90,91 | 75,7 - 98,1 | 0,7787 |
| ≤23,08 | 91,3 | 72,0 - 98,9 | 90,91 | 75,7 - 98,1 | 0,8221 |
| ≤24,63 | 95,65 | 78,1 - 99,9 | 90,91 | 75,7 - 98,1 | 0,8656 |
| ≤25,92 | 100 | 85,2 - 100,0 | 90,91 | 75,7 - 98,1 | 0,9091 |
| ≤27,97 | 100 | 85,2 - 100,0 | 87,88 | 71,8 - 96,6 | 0,8788 |
| ≤30,48 | 100 | 85,2 - 100,0 | 84,85 | 68,1 - 94,9 | 0,8485 |
| ≤31,72 | 100 | 85,2 - 100,0 | 81,82 | 64,5 - 93,0 | 0,8182 |
| ≤32,5 | 100 | 85,2 - 100,0 | 78,79 | 61,1 - 91,0 | 0,7879 |
| ≤35,94 | 100 | 85,2 - 100,0 | 75,76 | 57,7 - 88,9 | 0,7576 |
| ≤36,5 | 100 | 85,2 - 100,0 | 72,73 | 54,5 - 86,7 | 0,7273 |
| ≤38 | 100 | 85,2 - 100,0 | 69,7 | 51,3 - 84,4 | 0,697 |
| ≤38,7 | 100 | 85,2 - 100,0 | 66,67 | 48,2 - 82,0 | 0,6667 |
| ≤39,5 | 100 | 85,2 - 100,0 | 63,64 | 45,1 - 79,6 | 0,6364 |
| ≤40 | 100 | 85,2 - 100,0 | 60,61 | 42,1 - 77,1 | 0,6061 |
| ≤40,86 | 100 | 85,2 - 100,0 | 57,58 | 39,2 - 74,5 | 0,5758 |
| ≤41,82 | 100 | 85,2 - 100,0 | 54,55 | 36,4 - 71,9 | 0,5455 |
| ≤42,47 | 100 | 85,2 - 100,0 | 51,52 | 33,5 - 69,2 | 0,5152 |
| ≤44 | 100 | 85,2 - 100,0 | 48,48 | 30,8 - 66,5 | 0,4848 |
| ≤44,59 | 100 | 85,2 - 100,0 | 45,45 | 28,1 - 63,6 | 0,4545 |
| ≤45,33 | 100 | 85,2 - 100,0 | 42,42 | 25,5 - 60,8 | 0,4242 |
| ≤46,06 | 100 | 85,2 - 100,0 | 39,39 | 22,9 - 57,9 | 0,3939 |
| ≤46,95 | 100 | 85,2 - 100,0 | 36,36 | 20,4 - 54,9 | 0,3636 |
| ≤48,32 | 100 | 85,2 - 100,0 | 33,33 | 18,0 - 51,8 | 0,3333 |
| ≤51,01 | 100 | 85,2 - 100,0 | 30,3 | 15,6 - 48,7 | 0,303 |
| ≤53,01 | 100 | 85,2 - 100,0 | 27,27 | 13,3 - 45,5 | 0,2727 |
| ≤53,09 | 100 | 85,2 - 100,0 | 24,24 | 11,1 - 42,3 | 0,2424 |
| ≤53,73 | 100 | 85,2 - 100,0 | 21,21 | 9,0 - 38,9 | 0,2121 |
| ≤54,01 | 100 | 85,2 - 100,0 | 18,18 | 7,0 - 35,5 | 0,1818 |
| ≤56,01 | 100 | 85,2 - 100,0 | 15,15 | 5,1 - 31,9 | 0,1515 |
| ≤58,51 | 100 | 85,2 - 100,0 | 6,06 | 0,7 - 20,2 | 0,0606 |
| ≤80,01 | 100 | 85,2 - 100,0 | 3,03 | 0,08 - 15,8 | 0,0303 |
| ≤83,01 | 100 | 85,2 - 100,0 | 0 | 0,0 - 10,6 | 0 |

ROC: receiver operating characteristic.
